# Transcriptional and functional characterization of CD137L-dendritic cells identifies a novel dendritic cell phenotype

**DOI:** 10.1038/srep29712

**Published:** 2016-07-19

**Authors:** Zulkarnain Harfuddin, Bhushan Dharmadhikari, Siew Cheng Wong, Kaibo Duan, Michael Poidinger, Shaqireen Kwajah, Herbert Schwarz

**Affiliations:** 1 Department of Physiology, National University of Singapore, Singapore; 2 NUS Immunology Programme, Life Sciences Institute, National University of Singapore, Singapore; 3 NUS Graduate School for Integrative Sciences and Engineering, National University of Singapore, Singapore; 4 Singapore Immunology Network, Agency for Science, Technology and Research, Singapore

## Abstract

The importance of monocyte-derived dendritic cells (DCs) is evidenced by the fact that they are essential for the elimination of pathogens. Although *in vitro* DCs can be generated by treatment of monocytes with GM-CSF and IL-4, it is unknown what stimuli induce differentiation of DCs *in vivo*. CD137L-DCs are human monocyte-derived DC that are generated by CD137 ligand (CD137L) signaling. We demonstrate that the gene signature of *in vitro* generated CD137L-DCs is most similar to those of GM-CSF and IL-4-generated immature DCs and of macrophages. This is reminiscent of *in vivo* inflammatory DC which also have been reported to share gene signatures with monocyte-derived DCs and macrophages. Performing direct comparison of deposited human gene expression data with a CD137L-DC dataset revealed a significant enrichment of CD137L-DC signature genes in inflammatory *in vivo* DCs. In addition, surface marker expression and cytokine secretion by CD137L-DCs resemble closely those of inflammatory DCs. Further, CD137L-DCs express high levels of adhesion molecules, display strong attachment, and employ the adhesion molecule ALCAM to stimulate T cell proliferation. This study characterizes the gene expression profile of CD137L-DCs, and identifies significant similarities of CD137L-DCs with *in vivo* inflammatory monocyte-derived DCs and macrophages.

Monocyte-derived dendritic cells (moDC) are critical to a robust anti-pathogen immune response. During infection or inflammation, the tissue resident DCs are supplemented by moDC which efficiently capture and present antigens leading to a strong adaptive immune response^1^,^2^. *In vitro*, moDCs can be generated by treatment of monocytes with GM-CSF and IL-4, and are considered to represent *in vivo* DCs that appear during infection or inflammation^3^. However, murine studies have shown that GM-CSF is dispensable for development of moDCs *in vivo*^4^. A number of DC subsets have been identified in humans: blood DCs (BDCA1^+^ and BDCA3^+^ DCs), skin DCs, dermal CD14^+^ DCs and epidermal Langerhans cells^5^,^6^,^7^. A sub-set of epidermal DCs has also been shown to appear during atopic eczema with implications in skin inflammation^8^. These DC subsets have been reported to have considerably different gene expression profiles when compared to the moDCs generated *in vitro* by treatment with GM-CSF and IL-4 (referred to as cDC hence forth)^9^.

A number of clinical studies for treatment of cancer have been conducted to exploit the potential of DCs in inducing potent anti-tumour T cell responses, which could potentially kill tumour cells. Most clinical studies with DC vaccines or using DCs to expand antigen-specific T cells *in vitro* for adoptive transfer, have relied on the use of cDCs, and have employed various cytokine combinations for DC maturation^10^. Although a number of studies have shown objective clinical responses, the overall clinical benefits are low. These disappointing results are largely due the inability of cDCs to mount sufficiently strong T cell responses^11^,^12^. Thus, despite significant differences to *in vivo* DCs, GM-CSF and IL-4-derived cDCs remain the most widely used *in vitro* generated DC type that is used in immunological studies and for tumour immunotherapy.

CD137L-DCs are monocyte-derived DCs that are generated *in vitro* by inducing CD137 ligand (CD137L) signaling in peripheral human monocytes. CD137L is expressed by antigen presenting cells (APC), and APC use CD137L to costimulate CD137-expressing, activated T cells. The CD137 receptor/ligand system is capable of bidirectional signaling of which the molecular basis is that CD137L, just as CD137, is expressed as a transmembrane protein on the cell surface and can transmit a signal into the cell it is expressed on, a process referred to as reverse signaling^13^.

The CD137L signal is sufficient to drive monocyte to DC differentiation without the requirement of any exogenous cytokines. The CD137L-DCs have a different surface marker and cytokine profile compared to cDCs, and are several times more potent than cDCs in mounting antigen-specific T cell responses^14^,^15^,^16^.

Transcriptional studies have proven to be valuable in understanding the relationship between cell subsets. For instance, transcriptome analysis of primary DC subsets from man and mouse has helped to determine the extent of homology between the two species, and to classify conserved DC populations^9^,^17^.

In order to identify the molecular basis of the higher potency of CD137L-DC, we performed transcriptome analysis of CD137L-DCs and other *in vitro* generated APCs: immature cDCs (imm cDC), mature cDCs (mat cDC), macrophages, and recombinant Fc protein-treated monocytes (Fc-monocytes). Our data shows that the CD137L-DC transcriptome is closely related to both imm cDCs and macrophages. Genes involved in cell adhesion, lipid metabolism and the immune response are highly upregulated in CD137L-DCs, and elevated level of activated leukocyte cell adhesion molecule (ALCAM) contribute to T cell activation by CD137L-DCs. In addition, CD137L-DCs are also enriched in the gene signatures of *in vivo* BDCA1^+^ DCs, inflammatory DCs and macrophages. CD137L-DCs are thus a novel and highly potent *in vitro* DC type with a unique phenotype and similarities to *in vivo* APC.

## Results

### Multivariate transcriptome analysis indicates a close relationship of CD137L-DCs with *in vitro* generated immature cDCs and macrophages

In order to perform gene expression profiling, we generated CD137L-DCs from peripheral monocytes from 5 healthy donors together with the various control cells: imm cDC, mat cDC, macrophages, and Fc-monocytes. As an additional control, freshly isolated monocytes were used to denote the baseline. Total RNA was isolated after 7 days of culture, and the transcriptome of the six APC subsets was obtained using HumanHT-12 v4 Expression BeadChip arrays.

**Figure 1:**
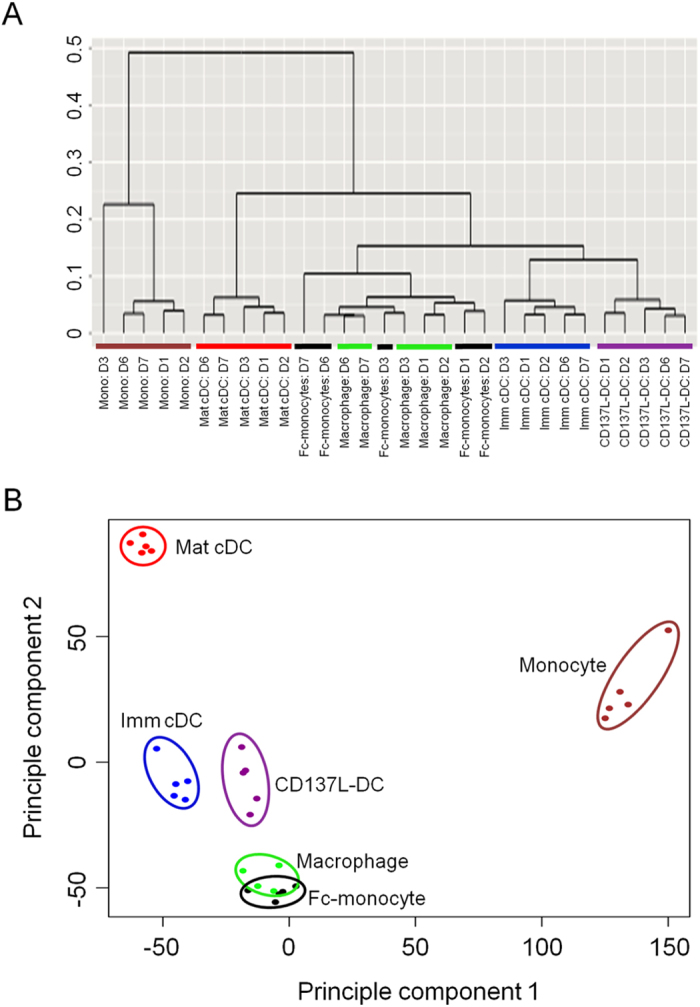
CD137L-DCs have a transcriptomic signature most similar to that of immature cDCs and macrophages. Monocytes from 5 healthy donors were differentiated to the various APC subsets. RNA from fresh monocytes and day 7 APC were used for gene expression profiling by Illumina Human HT12-V4 Expression BeadChip arrays. (**A**) Hierarchical clustering of genome wide expression profile based on Pearson linkage and (**B**) principal component analysis. The different APC populations are encircled and represented by different colours. Brown, green, blue, red, black and magenta represent the monocytes, macrophages, imm cDC, mat cDCs, Fc-treated monocytes, and CD137L-DCs, respectively. Principal components (PC) with maximum data variance were selected as representative axis. PC1 and PC2 represent 94% and 1.89% data variance, respectively.

To determine the relationship of CD137L-DCs with the other *in vitro* monocyte-derived APC, we performed multivariate analysis of the complete transcriptome dataset via hierarchical clustering (Fig. 1A) and principle component analysis (Fig. 1B). This analysis identified CD137L-DCs to be more closely related to imm cDCs and macrophages than to any other APC subset as the gene expression profile of CD137L-DCs clusters closer to both imm cDCs and macrophages. Thus, we conclude that the transcriptome profile of CD137L-DCs is unique and partly resembles the profiles of imm cDCs and macrophages.

**Figure 2:**
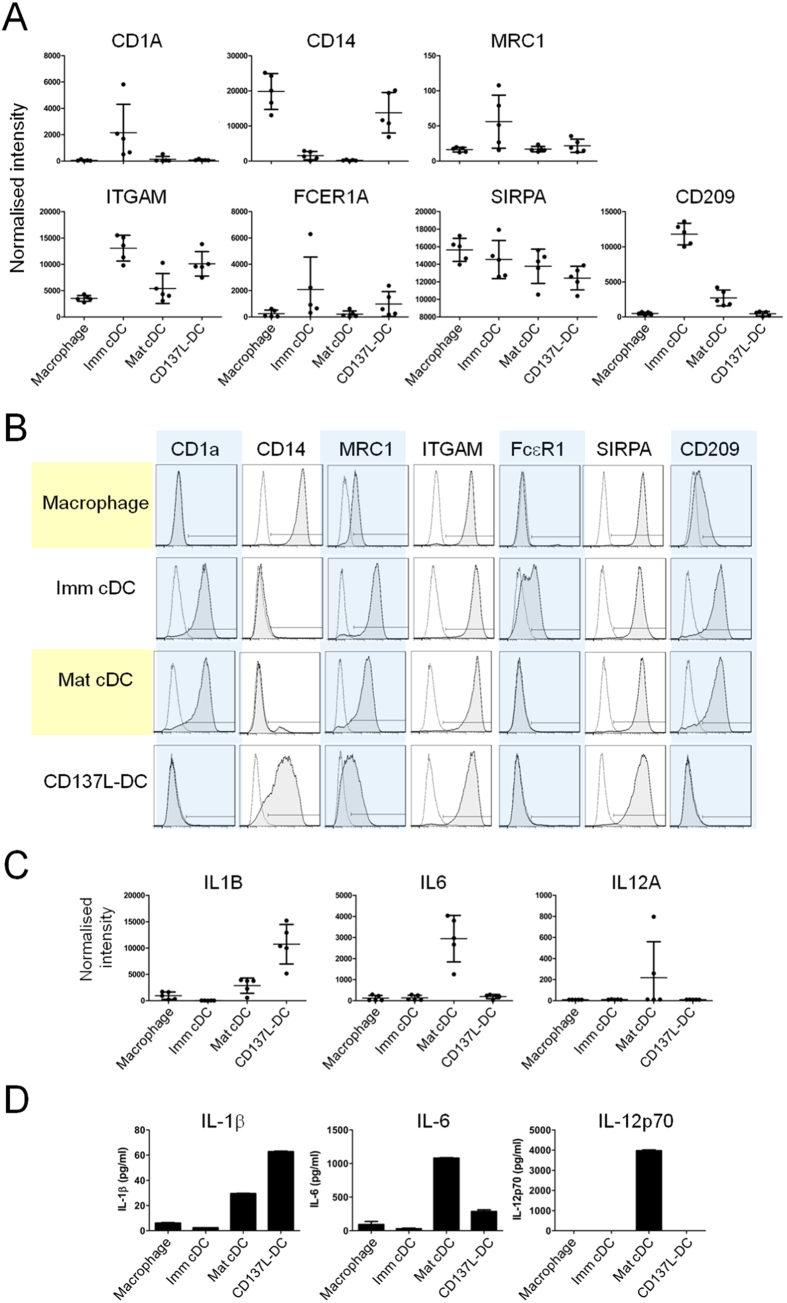
Validation of microarray data by protein expression analysis. Expressions of surface markers and cytokine release were used to validate results from microarray. (**A**) Microarray analysis denoting expression of genes encoding for surface proteins CD1a, CD14, MRC1, ITGAM, FcγR1A, SIRPA, and CD209, together with their corresponding (**B**) surface protein expression on macrophages, imm cDCs, mat cDCs, and CD137L-DCs analysed by flow cytometry. Flow cytometry histograms are representative of 3 independent experiments with comparable results. (**C**) Expression of genes encoding for cytokines IL-1β, IL-6 and IL-12A (p35), together with their corresponding (**D**) cytokine release detected by ELISA. Note that gene *IL12A* encodes for the IL-12p35 protein which is a component of the active heterodimer IL-12p70. Depicted are means ± standard deviation of triplicate measurements from one representative of 3 independent experiments.

To validate the microarray results, we generated the APC subsets (macrophages, imm cDC, mat cDC, and CD137L-DC) from monocytes of donors that were different from those used for the microarray. The expression of mRNA of the phenotypic markers CD1A, CD14, MRC1 (CD206), ITGAM (CD11b), FCER1A, SIRPA (CD172a) and CD209 (DC-SIGN) was compared across the APC subsets (Fig. 2A). The mRNA data could be confirmed at the protein level by flow cytometry since both data sets corresponded well with each other across all APC subsets (Fig. 2B). For instance, mRNA and surface protein expression of CD14 correlates across the APC subsets with high expression on macrophages and CD137L-DCs while it is virtually absent on cDCs. Likewise, CD1a is highly expressed at both mRNA and protein level by imm cDCs but not by macrophages and CD137L-DCs. Additionally, for the pro-inflammatory cytokines IL-1β and IL-6, and the Th1-polarizing cytokine IL-12p70, the protein expression (Fig. 2D) was comparable to the mRNA expression (Fig. 2C). As in the case of the surface markers, the relative expression of mRNA and protein tallied well with each other thus validating the microarray data.

### CD137L reverse signaling is responsible for the cDC-like properties of CD137L-DCs

The CD137L signal into APC is associated with activation, proliferation, expansion and differentiation^18^,^19^,^20^,^21^. It induces the differentiation of hematopoietic progenitors to the myeloid lineage giving rise to monocytes and macrophages^22^,^23^,^24^. *In vivo*, the CD137L signal drives infection induced myelopoiesis^25^ and the aging-associated shift of hematopoiesis from lymphopoiesis to myelopoiesis^26^. Further, it induces maturation of cDCs *in vitro*^27^,^28^,^29^.

As the CD137L-DC transcriptome is similar to those of *in vitro* generated imm cDCs and macrophages (Fig. 1), we wanted to determine if the CD137L reverse signaling contributes to cDC-like or macrophage-like properties or both, and whether Fc receptor (FcR) signaling induced by the Fc domain of the recombinant CD137-Fc protein plays a role in monocyte to CD137L-DC differentiation. In order to answer this question, we performed multivariate analysis of CD137L-DC signature genes and transcriptome of other *in vitro* generated APC populations.

We initially obtained differentially expressed genes (DEG) in CD137L-DCs by using the linear model for microarray data (limma) package, and paired sample analysis was applied on the datasets^30^. Gene probes were chosen with *p* values < 0.01, compared to those of control monocytes. The CD137L-DC signature probes were selected by obtaining the gene sets that were found in the intersection of DEG of CD137-Fc treatment vs monocytes, and DEG of CD137-Fc vs Fc-monocytes which eliminates DEG regulated by the Fc control protein (Supplementary Fig. S1). A total of 974 probes, corresponding to 829 genes, were identified as CD137L-DC signature genes. These genes thus represent the contribution of CD137L reverse signaling to the gene expression programme of CD137L-DCs.

**Figure 3:**
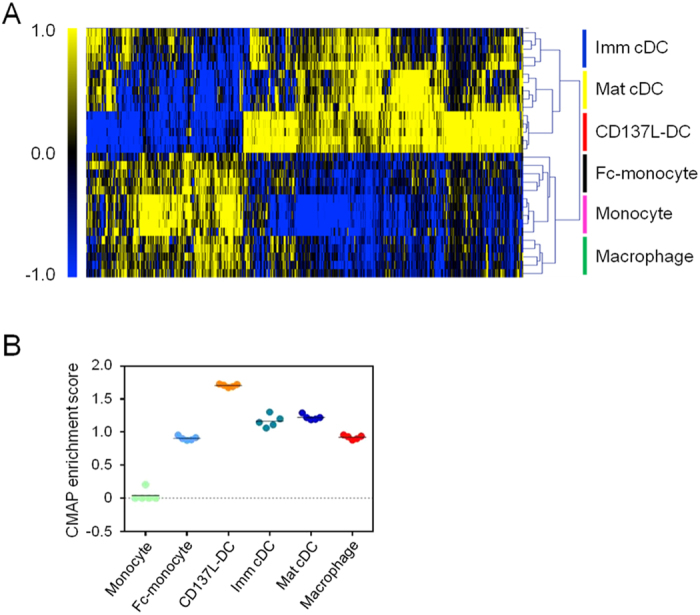
Cluster analysis of CD137L-DC signature genes with *in vitro* generated APC. (**A**) Hierachical clustering of the various APC subsets based on CD137L-DC signature genes (829 genes). Microarray profiles were obtained from five donors. (**B**) CMAP enrichment with CD137L-DC signature genes on all APC subsets, and with monocytes as the reference sample.

Hierarchical clustering and CMAP analysis (Fig. 3) of transcriptome datasets of *in vitro* generated APC populations and CD137L-DC signature genes indicated clustering of CD137L-DC signature gene sets with transcriptome datasets of mat cDCs and imm cDCs (Fig. 3A), and a higher enrichment of the CD137L-DC signature gene set in mat cDC and imm cDC transcriptome datasets (Fig. 3B). We therefore conclude that predominantly the CD137L signaling rather than the FcR signaling shifts the differentiation of monocytes to DC-like properties in CD137L-DCs. This conclusion is supported by the finding that blockade of FcR signaling does not affect the differentiation of CD137L-DCs (Supplementary Fig. S2).

### CD137L-DCs are highly adherent cells, with high ALCAM expression contributing to their potent T cell activation capacity

In order to identify the biological processes associated with the unique transcriptome of CD137L-DCs, we subjected the CD137L-DC up-regulated signature genes with >2 fold change, versus imm cDCs or mat cDCs, to Gene Ontology (GO) biologic process analysis using DAVID tools^31^. Categories with significant enrichment (*p* value < 0.05) were acquired and similar functional categories were grouped together (Table 1). GO enrichment of highly expressed DEG in CD137L-DCs compared to mat cDCs indicated that CD137L-DCs have higher activity in cell adhesion, locomotion and activation of immune responses. When CD137L-DCs were compared to imm cDCs, lipid processes, adhesion and immune responses were implied to be increased in CD137L-DCs. These differences in biological process-associated genes between CD137L-DCs and cDCs further confirm the unique phenotype of CD137L-DCs, and these differences may be responsible for the increased potency of CD137L-DCs.

Amongst the above-mentioned biological processes, the enrichment of genes associated with adhesion was of great significance for the study of CD137L-DCs since adhesion processes contribute to T cell activation, especially to the formation and stabilization of immune synapses between APC and T cells.

**Figure 4:**
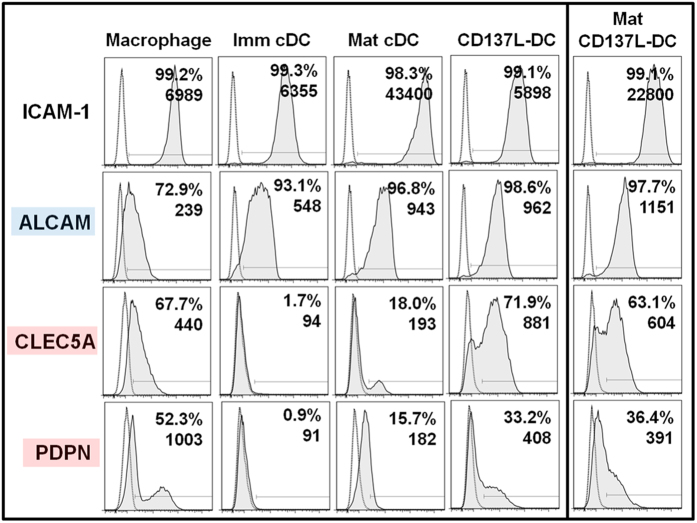
CD137L-DCs express adhesion molecules that are features of both macrophages and cDCs. DCs and APC were generated for 7 days and were treated for the final 18 h with LPS and IFN-γ to generate mat cDCs or R848 and IFN-γ to generate mat CD137L-DCs. Cells were harvested, immunostained for the indicated markers, and analysed by flow cytometry. ALCAM is predominantly expressed by cDCs while CLEC5A and PDPN are predominantly expressed by macrophages. Values in the histograms depict percentages and MFI of a single experiment. These data are representative of at least two independent experiments with comparable results.

Intracellular adhesion molecule 1 (ICAM-1) and lymphocyte function-associated antigen 1 (LFA-1) are found on APCs and T cells, respectively, and the engagement of these molecules is required for stable APC - T cell associations^32^,^33^. ICAM-1 was indeed expressed by all the APC types tested although cDCs expressed it at higher levels than CD137L-DCs (Fig. 4). Based on the DEG, we selected a panel of genes that are upregulated in CD137L-DCs, and are associated with cell adhesion or co-stimulation. Surface expression analysis revealed that adhesion-associated molecules such as ALCAM, C-type lectin domain family 5 member A (CLEC5A), and podoplanin (PDPN) are highly expressed by CD137L-DCs and mature (mat) CD137L-DCs (mat CD137L-DCs generated by treatment of CD137L-DC with R848 and IFN-γ for the final 18 h as described in Harfuddin *et al*.^14^) compared to cDCs (Fig. 4). Of note, CD137L-DCs express markers that are characteristic of macrophages (CLEC5A and PDPN), and of cDCs (ALCAM).

**Figure 5:**
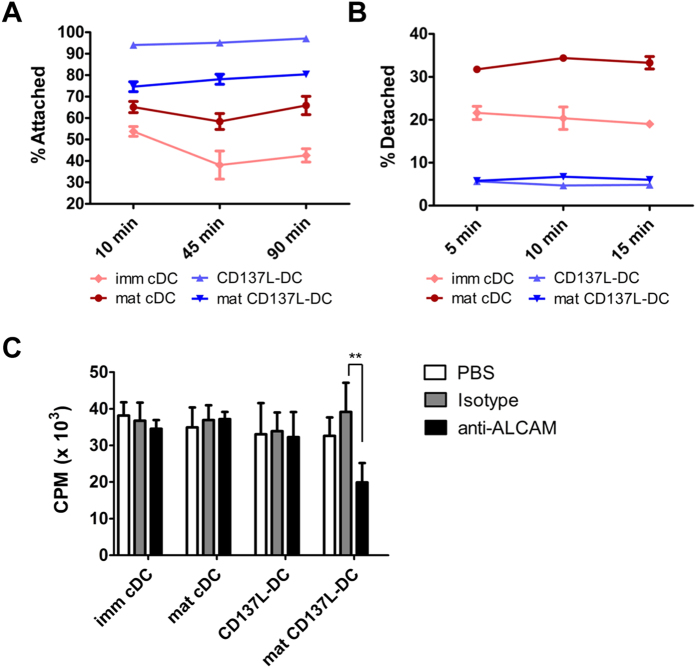
CD137L-DCs are highly adherent cells with ALCAM contributing to their increased T cell activation capacity. DCs were generated for a period of 7 days with cDCs matured by LPS and IFN-γ for the final 18 h while CD137L-DCs were matured by R848 and IFN-γ. Mat CD137L-DCs were previously described^14^. (**A**) DCs were seeded at equal densities on culture plate wells at 37 °C. After indicated time periods non-attached (i.e. non-adherent) cells were removed by washing. Thereafter, attached cells were harvested by treatment with tryspin-EDTA and flushing, and were then quantified using counting beads. Data shows percentages of attached cells with respect to the initial number of cells seeded at t = 0 min. Depicted are means ± standard deviations from duplicate samples. (**B**) DCs were incubated at 37 °C overnight before cells were treated with trypsin-EDTA for 5, 10 or 15 min. Detached DCs were harvested and counted using counting beads. Depicted are means ± standard deviations of duplicate samples of percentages of detached DCs. (**C**) DCs were co-cultured with allogeneic T cells at a ratio of 1:10 and treated with antagonistic anti-ALCAM antibody or isotype control. At day 4, proliferation was quantified by ^3^H-thymidine incorporation. Proliferation has been normalized to the PBS control (100%) and is expressed as means ± SD of triplicate measurements. *p < 0.05; **p < 0.01 (two-tailed, unpaired Student *t* test). This experiment is representative of 2 independent experiments with comparable results.

In order to determine whether the heightened expression of adhesion molecules is of functional significance, we tested the adhesion properties of CD137L-DCs. CD137L-DCs and cDCs were generated for 7 days, reseeded onto new plates and incubated for 10, 45 or 90 min before non-adherent cells were removed. Adherent cells were then harvested and quantified. More CD137L-DCs and mat CD137L-DCs than the imm or mat cDC remained attached to the plates at each time point indicating a faster attachment of CD137L-DCs (Fig. 5A). Next we tested whether CD137L-DCs not only attach faster but also adhere more strongly. APCs were allowed to adhere overnight, and were then treated with trypsin-EDTA for 5, 10 or 15 min to detach the cells. Both CD137L-DCs and mat CD137L-DCs displayed stronger adhesion as fewer than 10% of the cells had detached at each time-point while about 20–35% of imm cDCs or mat cDCs were detached (Fig. 5B).

In order to assess whether and how the elevated expression of adhesion molecules impacts the function of CD137L-DCs we investigated the influence of the heightened expression of ALCAM on T cell activation by CD137L-DCs. ALCAM expressed on DCs was previously reported to interact with CD6 on T cells, and this interaction promoted T cell proliferation^34^. We therefore measured proliferation of T cells upon their co-culture with the various DCs, and used antagonistic anti-ALCAM antibodies to block ALCAM - CD6 interaction. Interestingly, the presence of anti-ALCAM antibodies did not inhibit proliferation when T cells were co-cultured with either imm or mat cDCs (Fig. 5C). However, when T cells were stimulated by mat CD137L-DCs, blocking of ALCAM - CD6 interaction significantly reduced T cell proliferation, demonstrating the importance of ALCAM for T cell activation by CD137L-DCs.

### The CD137L-DC transcriptome is enriched for gene signatures of *in vivo* DCs and macrophages

In order to find out how CD137L-DCs compare to *in vivo* DCs, we determined the enrichment of the gene signatures of *in vivo* DCs and macrophages in the CD137L-DC transcriptome, and vice versa.

**Figure 6:**
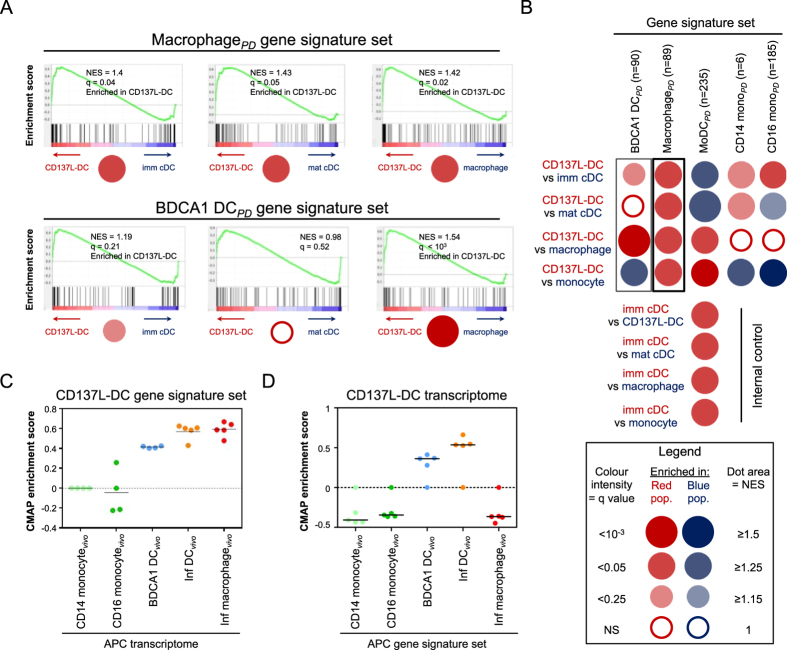
Gene set enrichment and connectivity map enrichment analyses of CD137L-DCs. GSEA of the gene signature (Segura *et al*.^35^) of blood and tonsil BDCA1^+^ DC (BDCA1 DC_*PD*_), alveolar macrophages (macrophage_*PD*_), monocyte-derived DC (MoDC_*PD*_), blood CD14^+^ monocytes (CD14 mono_*PD*_), and blood CD16 monocytes (CD16 mono_*PD*_) was performed. GSEA results for pairwise comparisons of APC populations; imm cDCs, mat cDCs, macrophages, and monocytes with CD137L-DCs. (**A**) The GSEA output is represented as a bar code characterised by two parameters; the normalized enrichment score (NES) and the false discovery rate statistical value (q). Green curve refers to the calculation of enrichment score. (**B**) Dot blot representation of pairwise GSEA comparison between CD137L-DCs with all other APC populations; imm cDCs, mat cDCs, macrophages, and monocytes. Gene signature sets comprise of a different number of genes (n). The dot colour corresponds to the font colour of the population in which the gene signature set is enriched. The dot area is proportional to the NES while the colour intensity is indicative of the false discovery rate statistical q value. CD137L-DC signature genes (829 genes) were derived from the formulation as described in Figure S1 and were subjected to CMAP analysis and GSEA. (**C**) CMAP enrichment analysis using CD137L-DC signature genes on *in vivo* APC samples acquired by Segura *et al*.^35^. CD14^+^ monocytes were used as the reference sample. (**D**) CMAP enrichment analysis using signature genes from *in vivo* APC samples on complete CD137L-DC transcriptome.

Segura *et al*.^35^ characterized the transcriptomes of human *in vivo* inflammatory DCs and inflammatory macrophages by determining in them the levels of enrichment of the gene signatures of human BDCA1^+^ DCs, macrophages, moDCs, CD14 monocytes and CD16 monocytes which were obtained from public databases. To unequivocally distinguish the cell types we added the suffix ‘*vivo*’ to *in vivo* APCs, while ‘PD’ refers to APC gene sets acquired from public databases by Segura *et al*. (Table 2). Our initial analysis of *in vitro* generated imm cDC, mat cDC, macrophages, Fc-monocytes and CD137L-DC identified similarities of CD137L-DC with both imDC and macrophages (Fig. 1). In order to confirm these data, and since the gene signatures that Segura and colleagues obtained from public databases represent not only *in vitro* but also *in vivo* DCs and macrophages, we tested the enrichment of these gene signatures in CD137L-DCs in a pairwise comparison via gene set enrichment analysis (GSEA). For each comparison, the data is presented as a barcode with a normalized enrichment score (NES) and a false discovery rate statistical q value (Fig. 6A). For an overview of the GSEA comparisons of all APC populations to CD137L-DCs, we represented each pairwise barcode output as a dot whose color corresponds to the cell type in which the particular gene signature is enriched. The size of the dot is proportional to the NES while a darker color corresponds to a lower q value (Fig. 6A,B). Compared to imm cDCs and mat cDCs, CD137L-DCs were enriched for the macrophage_*PD*_ gene signature. This was expected as the macrophage_*PD*_ gene signature is partly derived from *in vitro* generated monocyte-derived macrophages and the CD137L-DC transcriptome is related to *in vitro* generated monocyte-derived macrophages as well (Fig. 1). However, when compared to *in vitro* generated macrophages, the macrophage_*PD*_ gene signature was enriched in CD137L-DCs. This indicates that the CD137L-DC transcriptome also shares similarities with the transcriptome of *in vivo* macrophages as the macrophage_*PD*_ gene signature is partly contributed by the *in vivo* alveolar macrophages. Also, CD137L-DCs were more enriched for the gene signature of BDCA1^+^ DC_*PD*_ than macrophages or imm cDCs. Compared to mat cDCs, this BDCA1^+^ DC_*PD*_ gene signature was also enriched in CD137L-DCs, however, the enrichment did not reach statistical significance (NES = 0.98, q = 0.52). This corroborates the finding that the CD137L-DC transcriptome partly resembles those of DCs and macrophages (Fig. 1). CD137L-DCs were also enriched for the moDC_*PD*_ gene signature when compared to macrophages or monocytes. As imm cDCs, mat cDCs and moDC_*PD*_ were generated by GM-CSF and IL-4, the moDC_*PD*_ gene signature was predominantly enriched in imm cDCs and mat cDCs rather than in CD137L-DCs. Similarly, the monocyte_*PD*_ gene signature was enriched in monocytes than in CD137L-DCs. Further internal controls showed that the moDC_*PD*_ gene signature was enriched in imm cDCs than in any other APC type tested (Fig. 6B, bottom four red dots).

We then conducted a CMAP analysis with CD137L-DC signature genes on transcriptomes of inflammatory DC_*vivo*_, inflammatory macrophages_*vivo*_and other *in vivo* APCs acquired by Segura and colleagues^35^. CMAP analysis showed that CD137L-DC signature genes were enriched in inflammatory DC_*vivo*_, inflammatory macrophages_*vivo*_ and BDCA1^+^ DC_*vivo*_ (Fig. 6C) indicated by the positive enrichment scores. Conversely, we assessed whether signature genes of inflammatory DC_*vivo*_, inflammatory macrophages_*vivo*_ and BDCA1^+^ DC_*vivo*_ are enriched in the CD137L-DC transcriptome. We first identified gene signatures of the different cell types acquired by Segura and colleagues, and compared them to the complete CD137L-DC transcriptome dataset^35^. The inflammatory DC_*vivo*_ signature was clearly the dominant gene signature for CD137L-DCs suggesting a close relation between the two DC subsets (Fig. 6D). The enrichment for the BDCA1^+^ DC_*vivo*_ but not for the inflammatory macrophages_*vivo*_ gene signature provides further evidence that CD137L-DCs are indeed closely related to DC. Despite similarities, CD137L-DCs are not BDCA1^+^ DC as they do not express BDCA-1 (CD1c). Also, they only express low levels of CD141 (BDCA-3) and CD370 (Clec9a), (Supplementary Fig. S3).

In summary, comprehensive enrichment analysis of gene sets from various *in vivo* APCs indicates that at the transcriptional level, CD137L-DCs are more closely related to *in vivo* BDCA1^+^ DCs and inflammatory DCs than GM-CSF, IL-4 derived DCs. The fact that CD137L-DCs do not express BDCA-1 but have close similarity to BDCA-1^+^ DCs at the transcriptional level, indicates that only surface makers expression might not be sufficient to categorize DC subsets.

### CD137L-DCs produce IL-23 and IL-12p70 upon their activation

**Figure 7:**
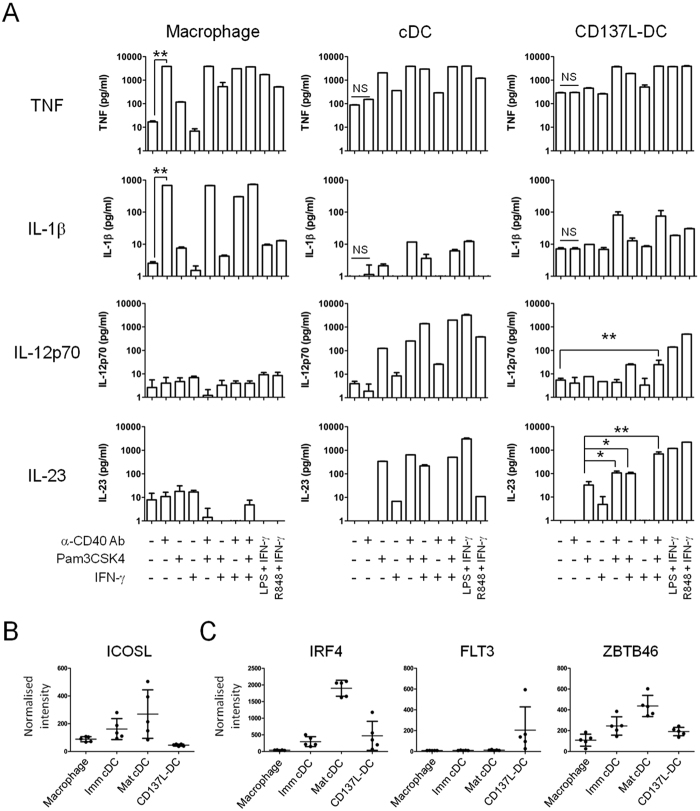
Activation of CD137L-DC with the combination of a TLR1/2 agonist, an agonistic anti-CD40 antibody and IFN-γ leads to the production of IL-23. (**A**) DC and macrophages were generated for 7 days and were then treated with the indicated factors (i.e. α-CD40 Ab, Pam3CSK4, and IFN-γ) for the final 24 h. Maturation with R848 and IFN-γ is the optimised maturation cocktail for CD137L-DC as described by Harfuddin *et al*.^14^. Supernatants were harvested and tested for the indicated cytokines by ELISA. Depicted are means ± standard deviation of triplicate measurements from a representative result of 2 independent experiments. NS = not significant, *P < 0.05, **P < 0.01 (two-tailed, unpaired Student *t* test). (**B**) Microarray data showing the expression of ICOSL gene. (**C**) Microarray data showing the expression of genes encoding growth factor receptors (IRF4 and FLT3) and the transcription factor (ZBTB46) associated with DC development.

Human inflammatory DC_*vivo*_, but not inflammatory macrophages_*vivo*_, from tumour ascites and synovial fluids of arthritis patients are able to secrete both IL-12p70 and IL-23 when activated with different combinations of Pam3CSK4 (a synthetic triacylated lipoprotein which is a TLR1/2 agonist), CD40L, and IFN-γ^35^. Similarly, *in vitro* generated macrophages do not produce substantial amounts of both cytokines, and treatment with Pam3CSK4, agonistic anti-CD40 antibody and IFN-γ failed to induce IL-12p70 or IL-23 (Fig. 7A). However, both cDCs and CD137L-DCs were able to produce high amounts of IL-23 upon activation. Pam3CSK4 alone was sufficient to trigger IL-23 secretion by cDCs, and addition of agonistic anti-CD40 antibody and/or IFN-γ was not required. Similarly, CD137L-DCs were able to produce IL-23 in the presence of Pam3CSK4 alone. However, secretion of IL-23 was increased when Pam3CSK4 was supplemented with anti-CD40 antibody and/or IFN-γ. For both cDCs and CD137L-DCs, Pam3CSK4 seems to be pivotal for the secretion of IL-23 although LPS can replace it for both cDCs and CD137L-DCs, or R848 for CD137L-DCs. Because CD137L-DCs do not express ICOS-L, which has been shown to be an alternative inducer for Th17 cell differentiation^36^, we propose that IL-23 is driving the IL-17 production in T cells as reported previously (Fig. 7B)^16^.

Likewise, IL-12p70 was produced by both cDCs and CD137L-DCs although the former were more potent in the production of this Th1-polarizing cytokine (Fig. 7A). Again, Pam3CSK4 alone was able to induce IL-12p70 production in cDCs but the addition of IFN-γ resulted in greater cytokine production. The combination of both factors is crucial for the IL-12p70 secretion by CD137L-DCs as neither Pam3CSK4 nor IFN-γ alone was able to induce the release of IL-12p70. Notably, upon engagement of CD40 the secretion of the inflammatory cytokines TNF and IL-1β was increased by macrophages, but not by cDCs and CD137L-DCs.

These data indicate that CD137L-DCs are closer to cDCs than to macrophages in their mode of activation and their secreted cytokine pattern. The functional similarity of CD137L-DCs with cDCs was expected as CD137L-DCs express mRNAs encoding DC-associated transcription factors such as IRF-4 and ZBTB46 at similar levels as imm cDCs (Fig. 7C)^37^. Furthermore, FLT3, a transcription factor associated with the DC lineage is highly expressed by CD137L-DCs but not by the other APC tested^38^,^39^. A salient observation is the resemblance in the cytokine profile of CD137L-DC to inflammatory DC_*vivo*_^35^.

## Discussion

Numerous DC-based immunotherapy trials for treatment of cancer(s) have been carried out with monocyte-derived DCs that were generated *in vitro* using GM-CSF and IL-4. Despite various antigen loading and maturation methods employed, the patient response rate is low with minimal survival benefits^11^,^12^. A major concern in this regard has been the fact that GM-CSF and IL-4 derived DCs differ significantly from their *in vivo* counterparts, and thus may lack the necessary co-stimulatory ability to mount a sufficiently strong immune response. For instance, a recent study by Helft *et al*.^40^ showed that GM-CSF-based differentiation of murine bone marrow cells leads to a heterogeneous population of DCs and macrophages that does not mimic *in vivo* DCs. It is therefore hoped that other types of DCs, that are ideally more potent than GM-CSF and IL-4 derived DCs, will fulfill the promises of DC-based cancer therapies.

Others and we have previously shown that CD137L reverse signaling *in vitro* differentiates human monocytes to potent DCs (CD137L-DCs) that are capable of stimulating T cells more effectively than GM-CSF and IL-4 derived DCs^14^,^15^,^16^. CD137L-DCs do not express common conventional DC markers such as CD1a and DC-SIGN, and have a lower MHC class II expression^16^. In the current study, we report that CD137L-DCs have a unique phenotype, differ from the GM-CSF and IL-4 derived DCs, and have significant similarities to *in vivo* inflammatory APCs. The CD137L-DC transcriptome displays features of *in vitro* generated GM-CSF and IL-4 derived DCs as well as of *in vitro* generated macrophages as evidenced by CLEC5A and podoplanin expression. Displaying features of both monocyte-derived DCs and macrophages has also been found to be a characteristic of *in vivo* inflammatory DCs^35^. Theoretically, the observed phenotype of CD137L-DCs could be due to a combination of CD137L and FcγR signaling. However, we found FcγR signaling not being necessary for CD137L-DC generation.

Transcriptome analysis indicated that the biological processes associated with CD137L-DCs and GM-CSF and IL-4 derived DCs differ, with one of these differences being cell adhesion. CD137L-DCs are highly adherent and the strong adhesive properties may contribute to their superior T cell activation capacity as has previously been reported^14^,^15^,^16^. The durability and stability of the immune synapse is critical for the priming of T cell responses. Long-lasting immune synapses correlate with activation of T cells while shorter interactions correlate with induction of tolerance^41^. DC-associated adhesion molecules such as ICAM-1, LFA-3 and several integrins are prerequisites for supramolecular activating complex (SMAC) formation, which is essential for the initiation of immune synapses with T cells^33^,^42^. Surface expression of ICAM-1 was indeed detected on CD137L-DCs, and was further increased upon their maturation. Yet mature cDC expressed more ICAM-1 suggesting that this molecule is not the main contributor to the superior capacity of CD137L-DCs to activate T cells.

The adhesion molecule ALCAM (CD166) was found to be highly upregulated in CD137L-DCs and GM-CSF and IL-4 derived DCs although it is more prominent in the former. Several studies have described ALCAM interaction with CD6 on T cells to be essential for immune synapse stabilization and optimal T cell activation and proliferation^34^,^43^,^44^. Our study demonstrates that the blocking of ALCAM on CD137L-DCs, but not on GM-CSF and IL-4 derived DCs, reduced their capacity to activate T cells as evidenced by the reduction in T cell proliferation. Interestingly, the effect of ALCAM blockade is more prominent on matured CD137L-DCs than on untreated CD137L-DCs despite similar ALCAM expression by these two cell populations. This could be because of a more robust binding of ALCAM to its ligand on mature than on immature DCs, possibly due to avidity regulation which is a process where molecules are redistributed into clusters on the cell membrane^34^,^45^. Blocking of ALCAM on GM-CSF and IL-4 derived DCs did not impair their potential to activate T cells suggesting that the ALCAM function is dispensable for GM-CSF and IL-4 derived DCs. Based on our data and the above-cited literature we conclude that ALCAM is important for the superior capacity of CD137L-DCs to stimulate T cells.

Several types of DCs have been identified in humans: blood derived BDCA1^+^, BDCA3^+^ DCs, dermal CD14^+^ DCs and additional inflammatory DC subsets such as slan DC, Tip-DC and inflammatory dermal DC (IDEC)^46^. Our data shows that CD137L-DCs are more enriched for gene signatures of *in vivo* BDCA1^+^ DCs and *in vivo* inflammatory DCs, described by Segura *et al*.^35^, than GM-CSF and IL-4 derived DCs. Also, the *in vivo* inflammatory DC and BDCA1^+^ DC transcriptome is enriched for the CD137L-DC gene signature indicating close similarities of CD137L-DCs to these *in vivo* DC subsets. Furthermore, inflammatory DCs are able to polarize naive T cells towards a Th17 phenotype via the production of cytokines such as IL-23, and not via ICOS-L stimulation^35^. We have shown previously that CD137L-DCs are superior activators of IL-17-producing T cells^16^. Our current data reveal that both GM-CSF and IL-4 derived DCs and CD137L-DCs, unlike macrophages, are able to produce IL-23 upon activation by TLR1/2 agonists, a characteristic also observed in inflammatory *in vivo* DCs. Furthermore, CD137L-DCs, but not GM-CSF and IL-4 derived DCs, transcribe only negligible levels of ICOS-L which is also the case for the inflammatory *in vivo* DCs^35^. These findings further indicate a functional similarity of CD137L-DCs to inflammatory *in vivo* DCs.

Just as inflammatory *in vivo* DCs, CD137L-DCs are a heterogeneous population. Individual CD137L-DC differ in expression of cell surface markers (CD14, ITGAM (CD11b), SIRPA (CD172a), etc) and cytokine levels (IL-1β). CD137L-DC were generated from negatively selected monocytes which already consist of different subpopulations. It is therefore not surprising that these different precursors differentiate to different APC.

Transcriptional and functional similarities of *in vivo* inflammatory DCs with CD137L-DCs suggests that the engagement of CD137L on infiltrating monocytes during inflammation could serve as a physiological stimulus for their differentiation to inflammatory DCs. Since CD137 is only expressed at sites of inflammation it would be a suitable signal for the induction of inflammatory monocyte-derived DC. Potential sources of CD137 required for initiating reverse CD137L signaling in monocytes would be activated T cells and also vascular endothelial cells at the site of inflammation^47^,^48^. The crosslinking of CD137L on monocytes induces M-CSF production, and in an autocrine manner M-CSF significantly prolongs the survival of monocytes *in vitro*^49^. Thus, the induction of CD137L signaling may provide both survival and differentiation signals for the development of inflammatory DCs *in vivo*. However, CD137L signaling is unlikely the sole factor required for the differentiation of monocytes to inflammatory DCs. While the surface expression of CD14, CD206, ITGAM (CD11b), SIRPA (CD172a) and CD209 on CD137L-DCs matches the phenotype of inflammatory DCs^35^, the expression of CD1a and FcεRI is different. However, various types of inflammatory DCs exist *in vivo*, which likely result from different combinations of stimuli at the local microenvironment^46^,^50^.

The main advantage of CD137L-DCs is their superior activity in priming and stimulating T cells. In addition, CD137L-DCs are induced by a signal (CD137) that is present exclusively at sites of inflammation or ongoing immune responses while it is uncertain whether high concentrations of GM-CSF and IL-4 occur naturally *in vivo*. Indeed, the natural differentiation signal may be a, or the, reason why CD137L-DCs resemble inflammatory *in vivo* DCs. Further, CD137L-DCs are easier to generate, since only one signal, a CD137L agonist, is required.

It is worth pointing out that not only reverse signaling by CD137L into monocytes enhances immune responses by inducing their differentiation to inflammatory DCs but also forward signaling by CD137 into T cells and NK cells^13^. CD137 agonists potently enhance immune responses, and are being evaluated for tumour immunotherapy^51^,^52^,^53^. Also, the inclusion of the cytoplasmic domain of CD137 into chimeric antigen receptors greatly increases their potency^54^,^55^. This evidence substantiates that CD137 – CD137L interaction is a powerful proinflammatory force^56^.

In summary, we demonstrate by gene expression and functional analyses that CD137L-DCs are highly similar to *in vivo* inflammatory DCs. This and the fact that CD137L-DCs have been shown to stimulate proliferation, IFN-γ secretion and the cytolytic activity of T cells more potently than GM-CSF and IL-4 derived DCs^14^,^15^,^16^, advocate the evaluation of CD137L-DCs as vaccines in human tumour immunotherapy.

## Materials and Methods

### Recombinant proteins and reagents

Recombinant human CD137-Fc fusion protein was purchased from R&D Systems (Minneapolis, MN, USA) while human IgG1 Fc fragment was purchased from Millipore (Billerica, MA, USA). GM-CSF, M-CSF and IL-4 were purchased from Peprotech (Rocky Hill, NJ, USA). IFN-γ was obtained from R&D Systems. Lipopolysaccharide (LPS) derived from *E. coli* of serotype 0111:B4 was obtained from Sigma-Aldrich (St. Louis, MO, USA). Resiquimod (R848) was purchased from Invivogen (San Diego, CA, USA).

### Generation of *in vitro* APCs

All blood samples in this study were obtained from the National University Hospital Blood Donation Centre under the approval by the Institutional Review Board, Singapore (IRB number: 13-079E) in accordance to the guidelines of the Health Sciences Authority of Singapore. Informed consent was obtained form all subjects. PBMC were prepared by Ficoll-Paque (GE Healthcare, Buckinghamshire, UK) density gradient centrifugation. Monocytes were isolated from PBMC by negative selection using the Monocyte Isolation Kit II (Miltenyi Biotec, Bergisch Gladbach, Germany) according to manufacturer’s instructions. Isolated monocytes were >95% pure based on antigenic phenotyping by CD14 staining.

To generate CD137L-DCs, monocytes were seeded onto polystyrene dishes (Becton Dickinson, Franklin Lakes, NJ, USA) pre-coated with 10 μg/ml of recombinant human CD137-Fc protein, in RPMI-1640 supplemented with 10% FBS, 50 μg/ml streptomycin and 50 IU/ml penicillin (R10 PS media) for 7 days. Maturation was induced during the last 18 h in the presence of R848 (1 μg/ml) + IFN-γ (50 ng/ml). To generate Fc-monocyte controls, monocytes were cultured on Fc-coated plates. cDCs were generated by culturing monocytes in R10 PS media in the presence of GM-CSF (80 ng/ml) and IL-4 (100 ng/ml) for 7 days. Cells were matured with LPS (1 μg/ml) + IFN-γ (50 ng/ml). Macrophages were generated by culturing monocytes in R10 PS media in the presence of M-CSF (100 ng/ml) for 7 days. Table 2 describes the cell types used in this study including the publically available transcriptomic datasets.

### Microarray processing and analysis

RNA was isolated from the generated APCs using Qiagen RNeasy micro kit (Qiagen, Hilden, Germany). RNA quality was confirmed using the Agilent Bioanalyzer (Agilent Technologies, Santa Carla, CA, USA). A total of 300 ng of total RNA was processed using the Illumina TotalPrep RNA Amplification Kit. Hybridization was performed on Illumina Human HT12v4 BeadChips. A total of 30 expression profiles were obtained, from the 6 APC subsets of 5 different donors (data deposited in GEO, accession number GSE60199). Readings were directly exported from the Illumina BeadStudio, and normalized using the procedures as described recently^57^. Differential expression analysis was done with the Limma Bioconductor package and the differentially expressed genes were selected with Benjamini-Hochberg multiple testing correction adjusted p-value < 0.05. Statistical analysis of the transcriptome data was performed using the R package (R Development Core Team). The ‘stats’ and ‘ggdendro’ packages were used for principal component analysis and hierarchical clustering, respectively.

To derive gene signatures of the *in vivo* derived APCs previously described by Segura *et al*. the deposited dataset (GEO, accession number GSE40484) was acquired and subjected to direct comparison of one cell type to all other cell types using t test^35^. Genes with adjusted p values < 0.05 and a fold-change >2 were selected as signature genes. The complete transcriptome of CD137L-DCs was then compared for the expression of these signature genes.

### Gene Set Enrichment Analysis

The Gene Set Enrichment Analysis (GSEA) was performed, as described by Subramanian and colleagues^58^ using the GSEA software v2.0 (http://www.broadinstitute.org/gsea). GSEA generates a ranked list of the significantly expressed genes from the expression data of the two populations being compared. The barcode represent the ranked list of genes for the two populations being tested with each end representing genes that are highly enriched in respective populations.

In all the analysis performed, the 5 gene sets originally derived from the public domain database (BDCA1 DC_*PD*_, macrophage_*PD*_, monocyte-derived DC_*PD*_, CD16 monocyte_*PD*_ and CD14 monocyte_*PD*_) as described by Segura *et al*.^35^ were used to determine their enrichment in CD137L-DCs compared to the other APC populations used in this study. For all gene sets, 1000 permutations with ‘phenotype’ algorithm were used. Normalized enrichment score (NES) >1 with false discover rate (FDR/q) < 0.25 was considered a significant enrichment as per the GSEA recommendations.

### Connectivity Map Analysis

Connectivity map (CMAP) analysis was performed as described by Lamb and colleagues^59^. The CMAP analysis works with a set of up- and down-regulated genes and generates enrichment scores which are scaled dimensionless quantities and measure the degree of enrichment of the gene profile in the sample tested. The enrichment scores indicate the relative similarity of the tested samples to the gene profile.

### Flow cytometry

Surface marker expression was determined using antibodies listed in Supplemental Table S1. To prevent non-specific binding, cells were blocked with FcR blocking reagent (Miltenyi Biotec), and were then stained with specific antibodies in PBS containing 0.5% bovine serum albumin (BSA) and 0.1% sodium azide (staining buffer) for 45 min at 4 °C in the dark. Unsatined samples were used as a control. Labeled cells were analyzed on a BD LSRFortessa or BD LSRFortessa X-20 (BD Bioscience, Franklin Lakes, New Jersey). Data were analyzed with FlowJo (Tree Star, Ashland, OR, USA).

### Cytokine production

CD137L-DCs, cDCs and macrophages were generated from monocytes as previously described. Day 6 cells were harvested and seeded into 48-well plates in duplicates containing their respective original supernatants. In certain conditions, plates were pre-coated with agonistic anti-CD40 antibody (clone 5C3, eBioscience, San Diego, CA, USA) at 10 μg/ml overnight prior to cell seeding. Pam3CSK4 (Invivogen) was added at 2 μg/ml while IFN-γ (R&D Systems) was added at 1000 IU/ml. After 24 h, supernatants were harvested and levels of IL-12p70 and IL-23 in supernatants were assessed by ELISA using the Duoset (R&D Systems) and Ready-SET-Go!^®^ Set (eBioscience), respectively. For ELISA-based validation of microarray data, IL-1β and IL-6 levels in supernatants were assessed using the Duoset (R&D Systems).

### Mixed-lymphocyte reaction

Day 6 CD137L-DCs and cDCs were activated by adding R848 (1 μg/ml) +IFN-γ (50 ng/ml) and LPS (1 μg/ml) + IFN-γ (50 ng/ml), respectively, for 18 h. APC were harvested and resuspended in fresh R10 PS medium at 2 × 10^5^ cells/ml. 50 μl of DC suspension was added into each well of a 96-well round bottom plate. Allogeneic T cells were added to give a final DC:T-cell ratio of 1:10 in total volume of 100 μl/well. Antagonistic anti-ALCAM Ab (clone 105901, R&D Systems) or control mouse IgG1 isotype (clone MOPC-21, Sigma-Aldrich) were added at 10 μg/ml. Culture plates were incubated for a total of 4 days and 0.5 μCi of ^3^H-thymidine (PerkinElmer, Boston, MA) per well were added for the final 18 h of culture. Counts were assessed by TopCount (PerkinElmer) liquid scintillation and luminescence counter.

### Attachment/detachment assay

Day 7 APC were incubated with 10 mM EDTA/non-enzymatic cell dissociation medium followed by cell scraping for complete cell harvest. Cells were resuspended in R10PS and seeded into individual wells of a 48-well plate at 10^5^ cells/well. For attachment assay, culture plate was centrifuged and incubated at 37 °C for the indicated time-periods. Supernatants containing suspended cells were gently collected, washed with PBS without flushing, and were then discarded. Complete harvest of the still adhered cells was done by treatment with trypsin-EDTA and continuous flushing. These cells were labelled as ‘attached cells’. For detachment assay, culture plate was centrifuged and was incubated overnight at 37 °C. Cells were detached by treatment with typsin-EDTA for the indicated time-points. Cells were harvested followed a gentle wash with PBS. Trypsin-EDTA and washing PBS were pooled and labelled as ‘detached cells’. The absolute number of cells was counted using CountBrightTM absolute counting beads. Propidium iodide (PI) was added for dead cell exclusion.

### Statistical analysis

Statistical significance was determined by a two-tailed unpaired Student’s t-test.

## Additional Information

**How to cite this article**: Harfuddin, Z. *et al*. Transcriptional and functional characterization of CD137L-dendritic cells identifies a novel dendritic cell phenotype. *Sci. Rep.* **6**, 29712; doi: 10.1038/srep29712 (2016).

## Supplementary Material

Supplementary Information

## Figures and Tables

**Table 1:** GO enrichment of biologic processes of CD137L DEGs compared to other APC.

Term	Count	*p*
CD137L-DC differentially expressed genes with ≥2× fold-change compared to imm cDC		
*Immune response*		
GO:0009611: Response to wounding	23	1.2E-6
GO:0006954: Inflammatory response	12	3.2E-3
GO:0006952: Defence response	15	2.7E-2
*Lipid processes*		
GO:0010743: Regulation of foam cell differentiation	5	2.7E-4
GO:0010885: Regulation of cholesterol storage	4	3.2E-4
GO:0010883: Regulation of lipid storage	4	2.3E-3
*Adhesion*		
GO:0007155: Cell adhesion	22	2.6E-4
GO:0022610: Biological adhesion	22	2.6E-4
CD137L-DC differentially expressed genes with ≥2× fold-change compared to mat cDC		
*Adhesion*		
GO:0007155: cell adhesion	25	1.1E-5
GO:0022610: biological adhesion	25	1.1E-5
*Locomotion*		
GO:0042330: taxis	10	2.2E-4
GO:0006935: chemotaxis	10	2.2E-4
GO:0007610: behaviour	16	1.1E-3
GO:0007626: locomotory behaviour	11	2.9E-3
*Immune response*		
GO:0009611: response to wounding	21	1.6E-5
GO:0006954: inflammatory response	10	2.5E-2
GO:0006952: defence response	13	9.9E-2

Shown are representative biologic processes from the top 7 enrichment clusters obtained from ‘functional annotation clustering’ from DAVID Bioinformatics Resources 6.7. Categories from ‘GOTERM_BP_FAT’ were used.

**Table 2:** Nomenclature of cell types described in this study.

*In vitro*-generated APC: Harfuddin *et al*.^14^		
	*Type of APC*	*Classification*
**1**	Monocytes	Blood CD14^+^ monocytes
**2**	Macrophages	M-CSF generated
**3**	Immature conventional DC	GM-CSF, IL-4 generated
**4**	Mature conventional DC	GM-CSF, IL-4, + LPS, IFN-γ generated
**5**	Fc-monocytes	Fc protein generated
**6**	CD137L-DC	CD137-Fc protein generated
***In vivo*-derived APC: Segura** ***et al***.^35^		
	*Type of APC* [*vivo: in vivo*]	*Classification*
**1**	CD14 monocyte_*vivo*_	CD14^+^CD16^−^ monocytes
**2**	CD16 monocyte_*vivo*_	CD14^dim^CD16^+^ monocytes
**3**	BDCA1^+^ DC_*vivo*_	HLA-DR^+^CD11c^+^CD14^−^CD16^−^BDCA1^+^ cells from blood
**4**	Inflammatory DC_*vivo*_	HLA-DR^+^CD11c^+^CD16^−^BDCA1^+^FcεRI^+^ cells from inflammatory fluids/ascites
**5**	Inflammatory macrophage_*vivo*_	HLA-DR^+^CD11c^+^CD16^+^BDCA1^−^FcεRI^−^ cells from inflammatory fluids/ascites
**Gene set from public domain: Derived by Segura** ***et al***.^35^		
	*Type of APC* [*PD*: public domain]	*Classification*
**1**	CD14 monocyte_*PD*_	Blood CD14^+^ monocytes
**2**	CD16 monocyte_*PD*_	Blood CD16^+^ monocytes
**3**	Monocyte-derived DC_*PD*_	*In vitro* generated monocyte-derived DC
**4**	Macrophage_*PD*_	PBMC derived, monocyte derived, and alveolar macrophages
**5**	BDCA1 DC_*PD*_	Blood and tonsil BDCA1^+^ DC

The BDCA1^+^ DC gene signature is derived from an *in vivo* DC subset (BDCA1^+^ DC), while the macrophage gene signature is a combination derived from *in vitro* generated macrophage gene signatures and *in vivo* alveolar macrophage gene signature^3534^. The moDC gene signature is derived from *in vitro* monocyte-derived DCs that were generated by GM-CSF and IL-4. The CD14 and CD16 gene signatures are from blood CD14^+^ and CD16^+^ monocytes, respectively. Segura and colleagues acquired the transcriptome of *in vivo* inflammatory DCs and inflammatory macrophages that were isolated from the synovial fluid of rheumatoid arthritis patients and inflammatory tumour ascites, along with other *in vivo* APCs. Table 2 describes the cell populations used in this study. To unequivocally distinguish the cell types we add the suffix ‘*vivo’* to *in vivo* APCs, while ‘PD’ refers to APC gene sets acquired from public databases by Segura *et al*.^35^.
