# Supplementary material for: Transcriptional and functional characterization of CD137L-dendritic cells identifies a novel dendritic cell phenotype
Source: Sci Rep. 2016 Jul 19;6:29712. doi: 10.1038/srep29712 (PMC4949477; doi:10.1038/srep29712)

**Transcriptional and functional characterization of CD137L-dendritic cells identifies a novel dendritic cell phenotype**

**Zulkarnain Harfuddin, Bhushan Dharmadhikari, Siew Cheng Wong, Kaibo Duan, Michael Poidinger, Shaqireen Kwajah & Herbert Schwarz**


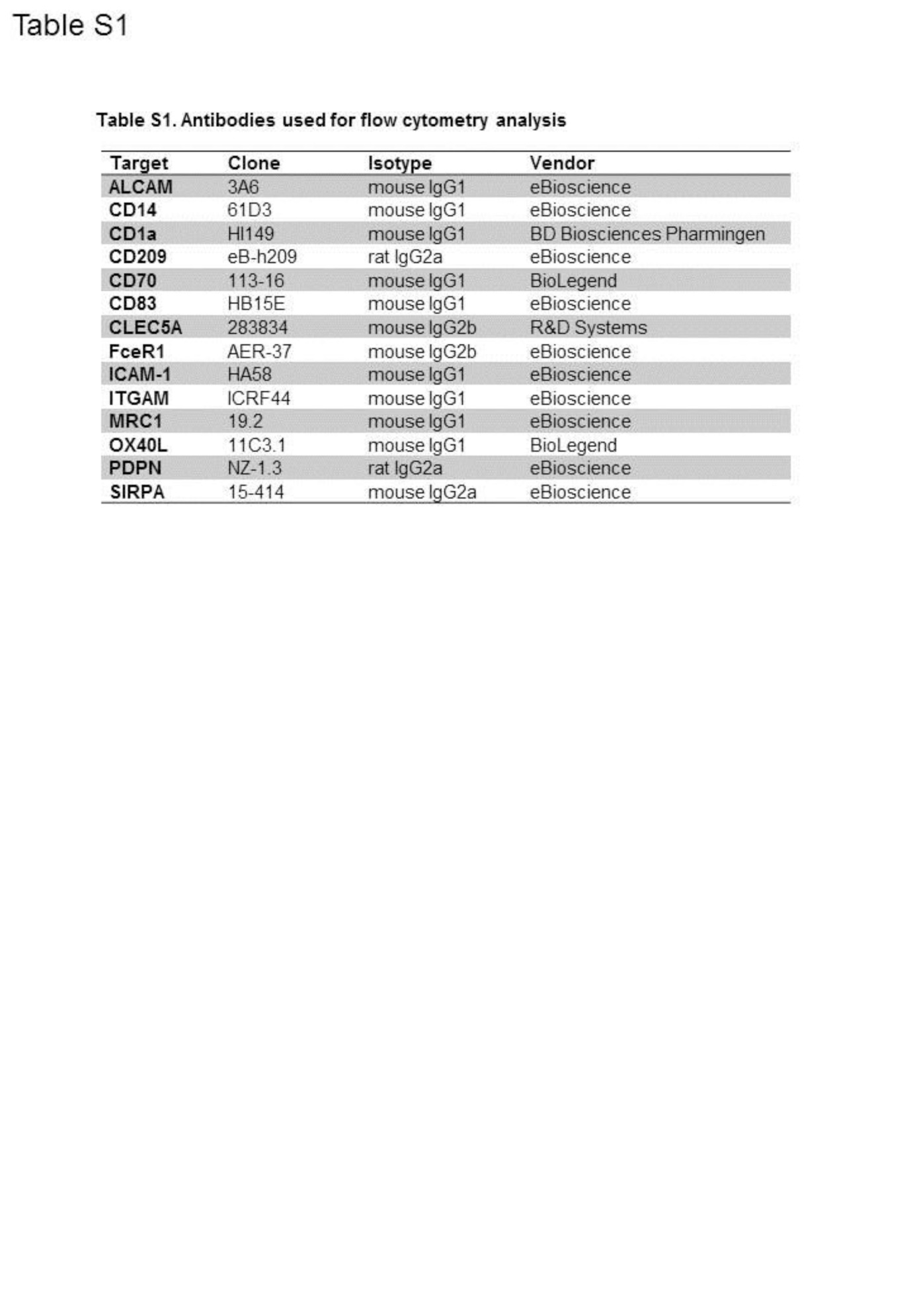


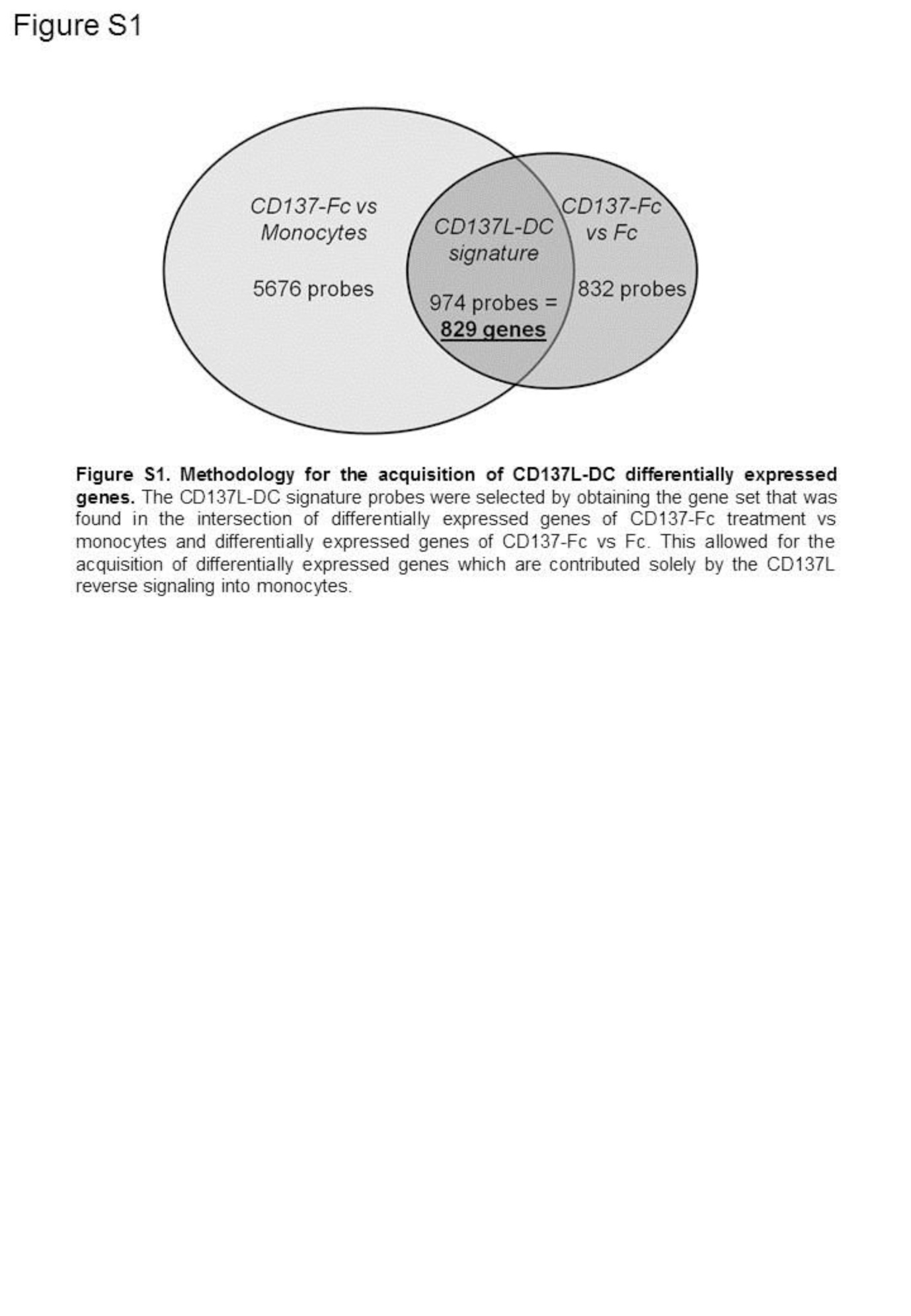

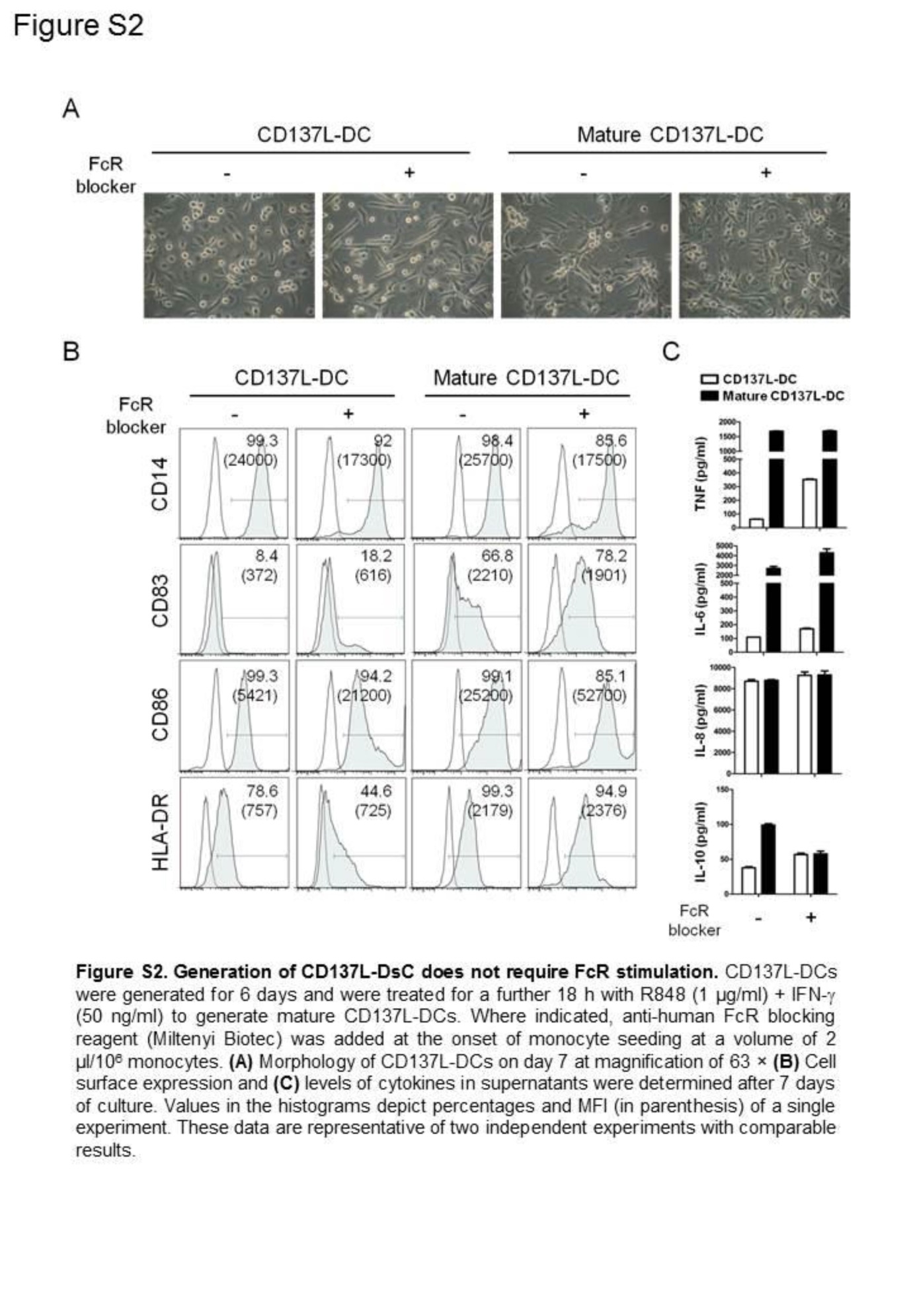

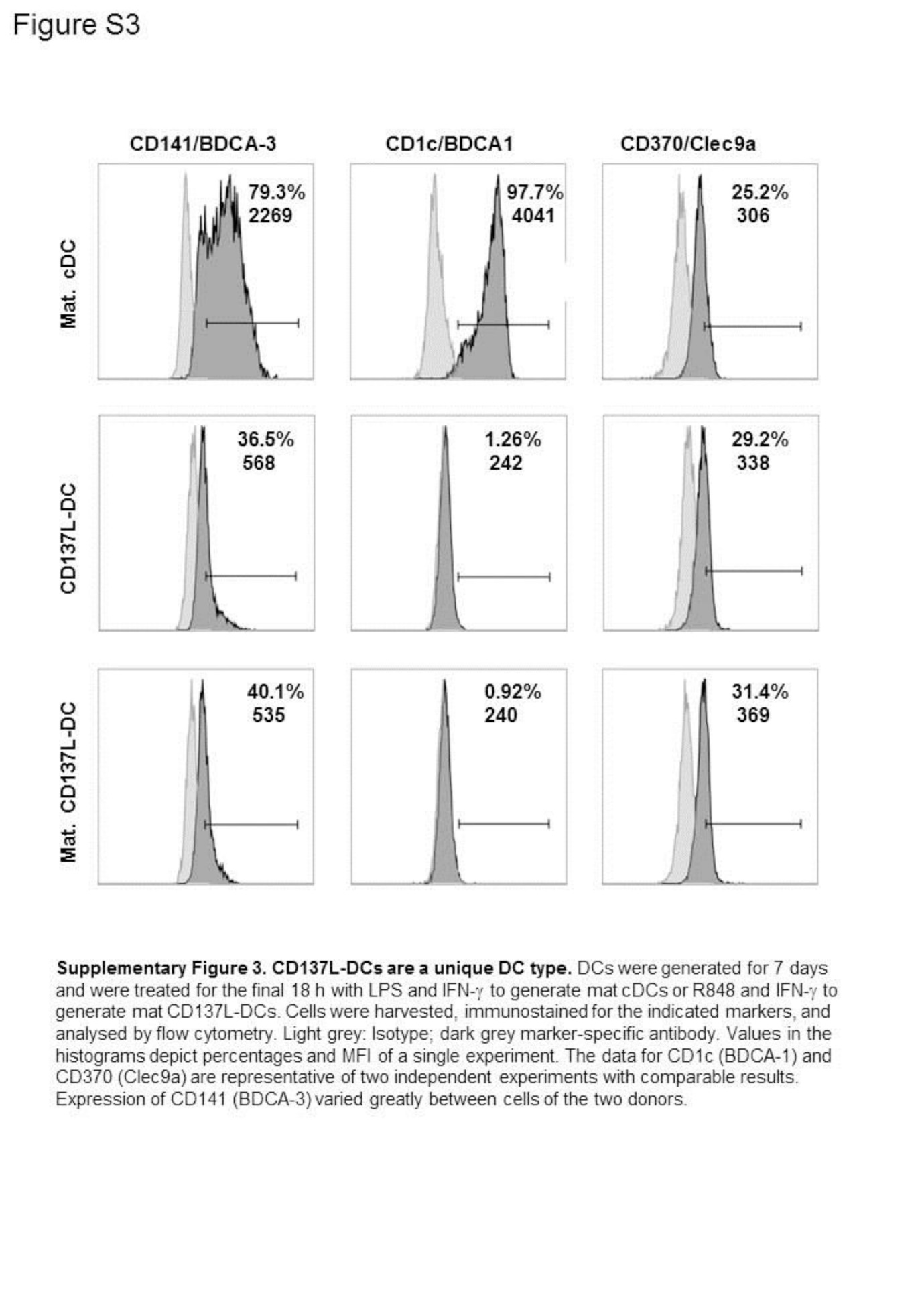

Supplement: Supplementary Information [file srep29712-s1.doc]
